# Effect of tolytoxin on tunneling nanotube formation and function

**DOI:** 10.1038/s41598-019-42161-6

**Published:** 2019-04-05

**Authors:** Aysegul Dilsizoglu Senol, Anna Pepe, Clara Grudina, Nathalie Sassoon, Ueoka Reiko, Luc Bousset, Ronald Melki, Jörn Piel, Muriel Gugger, Chiara Zurzolo

**Affiliations:** 1 Institut Pasteur, Unité Trafic Membranaire et Pathogenèse, 25–28 Rue du Docteur Roux, 75724 Paris CEDEX 15, France; 2 Institut Pasteur, Collection des Cyanobactéries, 25–28 Rue du Docteur Roux, 75724 Paris CEDEX 15, France; 3 Institute of Microbiology, Eigenössische Technische Hochschule (ETH) Zurich, Vladimir-Prelog-Weg 4, 8093 Zurich, Switzerland; 4 Paris-Saclay Institute of Neuroscience, CNRS, 1 Avenue de la Terrasse, 91190 Gif-sur-Yvette, France

## Abstract

Tunneling nanotubes (TNTs) are actin-containing membrane protrusions that play an essential role in long-range intercellular communication. They are involved in development of various diseases by allowing transfer of pathogens or protein aggregates as well as organelles such as mitochondria. Increase in TNT formation has been linked to many pathological conditions. Here we show that nM concentrations of tolytoxin, a cyanobacterial macrolide that targets actin by inhibition of its polymerization, significantly decrease the number of TNT-connected cells, as well as transfer of mitochondria and α-synuclein fibrils in two different cell lines of neuronal (SH-SY5Y) and epithelial (SW13) origin. As the cytoskeleton of the tested cell remain preserved, this macrolide could serve as a valuable tool for future therapies against diseases propagated by TNTs.

## Introduction

Tunneling nanotubes (TNTs) are actin-containing membranous protrusions that connect remote cells and emerged as a novel mechanism of cell-to-cell communication. Different from other cellular protrusions, they are straight with a small diameter (20–500 nm) and a length up to 100 μm. TNTs are not tethered to the substrate, but rather hovering in the culture medium^1^. They were shown to transfer signals and various cargos such as membrane proteins, soluble molecules, organelles, and thus implicated in several physiological processes^2–4^. They should also be open-ended to allow the transfer of these cargoes^5^. Moreover, these structures were reported to be hijacked by various pathogens such as viruses^6–8^, bacteria^9^, huntingtin^10^, prion^11–13^ and α-synuclein (α-syn)^14^ to spread from one cell to another.

Diverse cell types including epithelial, fibroblastic, immune and neuronal cells form TNTs *in vitro*^2,4^ and several studies have provided evidence of their existence *in vivo*^15–18^. Recently, TNTs have been identified in different types of cancer cells in which they were linked to cancer development^19–23^. Overall, these studies underlined the role of TNTs in many diseases from cancer to neurodegenerative disorders.

Blocking TNT formation has emerged as an approach for therapeutic targets^3,6,22,24^. Even though the molecular mechanism of how TNTs are formed is not yet clear, it has been shown that actin is the major cytoskeleton component of all TNTs^1^ and actin polymerization is required for their assembly^16,25,26^. In particular, our recent data demonstrate that both filopodia and TNTs relay on actin polymerization and use the same actin regulators, but in opposite manner, thus suggesting that TNTs and filopodia are different structures^27^. Studies on actin polymerization targeting compounds revealed inhibition of TNT formation at µM concentrations^1,9,11,22,28^; however, this is not specific and affects all the actin cytoskeleton.

Tolytoxin, a cyanobacterial macrolide, acts on actin filament dynamics^29^. It specifically disrupts microfilament organization of cells by inhibiting actin polymerization and inducing fragmentation of filamentous-actin (F-actin) while having no effect on microtubules or intermediate filaments *in vitro*^29^. Tolytoxin was shown to exert this function by binding to barbed end of F-actin and the corresponding region of globular actin (G-actin)^30^. The macrolide core moiety interact with the actin surface, whereas the “tail” region interferes with actin monomers, thus causing filament disruption^30^. Tolytoxin belongs to a large family of cytotoxic compounds with similar actin-binding properties^31^. Tolytoxin extracted from *Scytonema* sp. has been shown to have cytostatic effects in human epidermoid^29^, breast^32^ and ovarian carcinoma cells^33^ as well as to murine lymphocytic leukemia cells^29^ and to be particularly toxic. Recently, pure cultures of cyanobacteria producing tolytoxin were obtained, which allow to examine further these macrolide activities^34,35^.

In the present study, we investigated the effect of tolytoxin from two different cyanobacterial genera, *Scytonema* and *Planktothrix*, on TNT formation and function in both neuronal SH-SY5Y and non-neuronal SW13 cells. We report that minimal concentrations of tolytoxin at the nM range specifically act on TNT-like structure formation for both cell types and not on substratum attached filopodia. We also demonstrated that nM of tolytoxin decrease TNT-dependent transfer of cargoes from one cell to another. Given the contribution of TNT-mediated organelle and pathogen transfer in development of cancer and neurodegenerative diseases, tolytoxin could be a novel candidate in potential therapies for different diseases in which TNTs are involved.

## Results

### Characterization of tolytoxin of pure cyanobacteria on SW13 and SH-SY5Y cells

**Figure 1:**
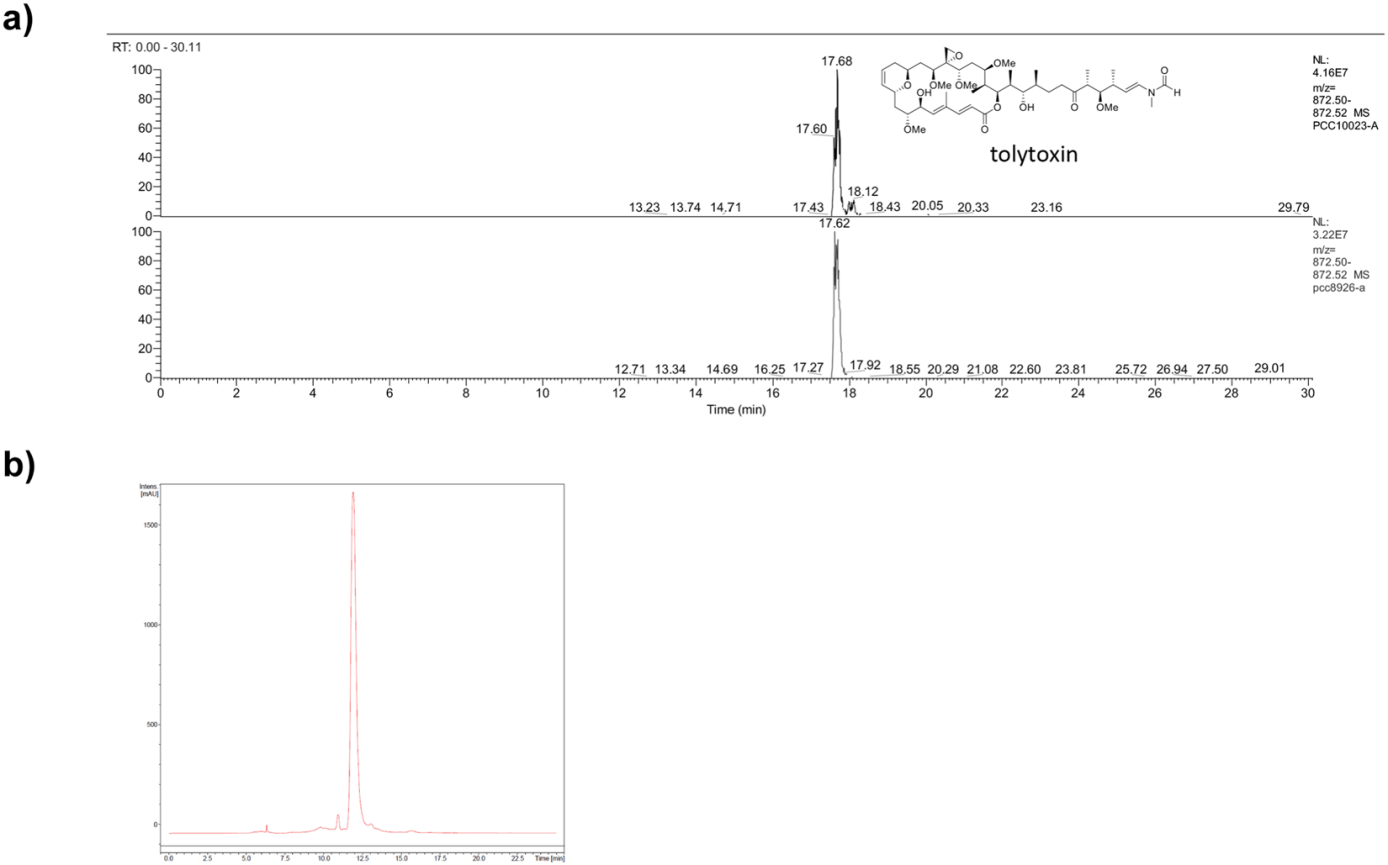
Characterization of tolytoxin produced by pure cyanobacteria. (**a)** HR-LCMS data of extracted ion chromatogram (*m*/*z* 872.50–872.52) of tolytoxin from *Scytonema* sp. PCC 10023 (upper, standard) and from *Planktothrix paucivesiculata* PCC 8926 (lower). (**b**) HPLC chart of the fraction containing pure tolytoxin from PCC 8926.

In order to test the specific effect of tolytoxin, we extracted pure tolytoxin from two cyanobacterial strains, *Planktothrix paucivesiculata* PCC 8926 and *Scytonema* sp. PCC 10023, and carried our analysis in two different cell lines, neuronal SH-SY5Y cells and adrenal gland/cortex SW13 cells. By genome mining, we identified a *trans*-acyltransferase polyketide synthase gene cluster in *Planktothrix paucivesiculata* PCC 8926 identical to the one previously revealed from *Scytonema* sp. PCC 10023^35^, along with various other natural product clusters for predicted terpenes and cyanobactins. The 93 kb-long sequence of this biosynthetic gene cluster is 98%, 91% and 88% similar to the tolytoxin/luminaolide B gene cluster of *Planktothrix* sp. PCC 11201, *Planktothrix paucivesiculata* PCC 9631, and *Scytonema* sp. PCC 10023, respectively (Supplementary Fig. S1a). Isolation and characterization of polyketides from PCC 8926 cultures revealed the presence of tolytoxin, but not of other congeners, such as scytophycins previously detected in *Scytonema* sp. PCC 10023 (Fig. 1a,b, Supplementary Fig. S1b,c).

**Figure 2:**
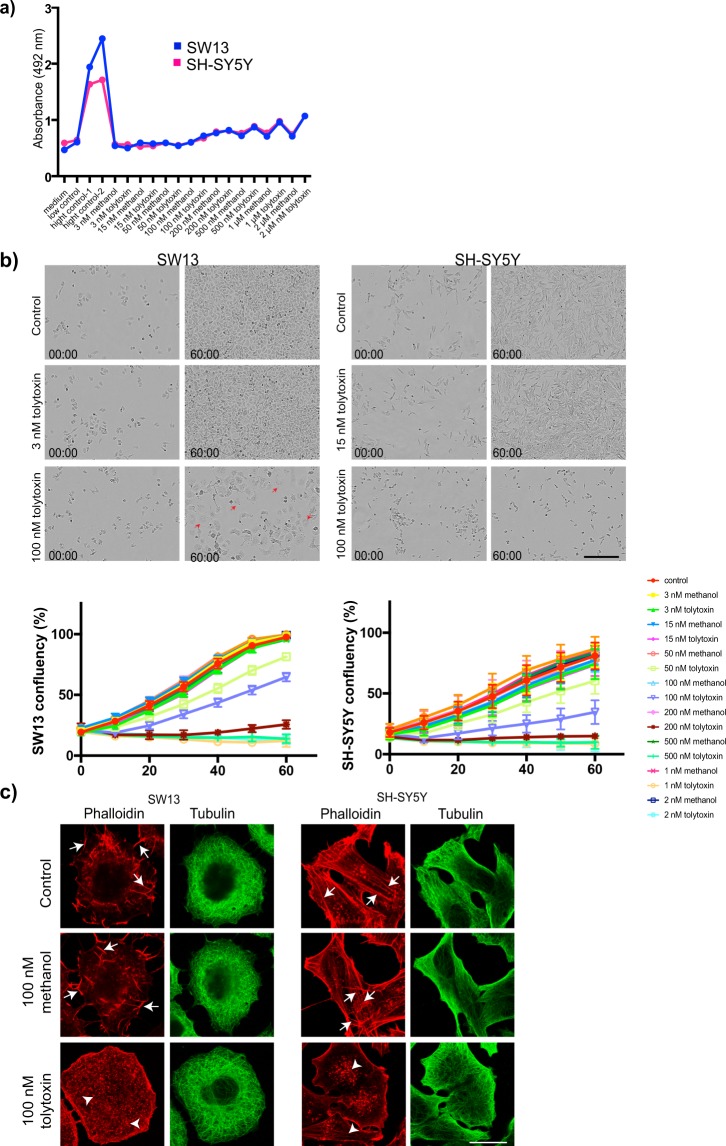
Effect of tolytoxin on cell viability, proliferation and cytoskeleton. (**a**) Lactate dehydrogenase release from three independent experiments are presented for SW13 and SH-SY5Y cells. medium: negative control, low control: LDH activity released from the untreated cells (spontaneous LDH release); high control-1: cells were lysed with 5 μl of lysis buffer; high control-2: cells were lysed with 10 μl of lysis buffer; tolytoxin and methanol treatments from 3 nM to 2 µM are indicated on the graph. (**b**) Cell confluency detection with Incucyte. Bright field images of control, 3 nM and 100 nM tolytoxin treated SW13 cells and control, 15 nM and 100 nM tolytoxin treated SH-SY5H cells at the beginning of acquisitions, time 0 (00:00), and at the end of acquisitions 60 h (60:00) are presented. Graphs showing the confluency of SW13 and SH-SY5Y cells over time in given concentrations of tolytoxin or methanol. Data were collected from two independent experiments. Scale bar: 150 µm. (**c**) Effect of tolytoxin on actin and microtubule cytoskeleton. Actin (rhodamine phalloidin) and microtubule (tubulin) labeling for SW13 (left) and SH-SY5Y cells (right) in control, 100 nM of tolytoxin or methanol treated cells. Arrows indicate actin filaments, arrowheads indicate disrupted actin filaments. One representative image is presented among four independent experiments. Scale bar: 10 µm.

First, we evaluated the effect of tolytoxin extracted from *Planktothrix paucivesiculata* PCC 8926 (referred as 8926 thereafter) on cell viability by lactate dehydrogenase (LDH) release assay. Briefly, SW13 and SH-SY5Y cells were treated with wide range of concentrations of tolytoxin (from 3 nM to 2 µM) for 18 h and LDH release in the medium was quantified. All experiments were performed in parallel with methanol treatment in the same concentration as used for dissolving tolytoxins (Me-control) and with untreated cells (Control). For both cell types, LDH release started to increase, in a dose dependent manner, from 100 nM of tolytoxin treatment. Me-Control also increased LDH release starting from 200 nM (Fig. 2a). Next, to evaluate the effect of tolytoxin on cell division, both cell types were plated on B12 well plates and incubated 24 h. Cells were then treated with same concentration range used in LDH release experiments and immediately started to be monitored during 60 h by Incucyte ZOOM cell imaging system which automatically acquired images from each condition in every 30 min. Then, cell confluency was quantified for all conditions. For both cell types, cell proliferation started to be affected at 50 nM of tolytoxin and a clear cytostatic effect was observed at 100 nM of tolytoxin, which increased in a dose-dependent manner (Fig. 2b). In addition, polynucleated cells were detectable in SW13 cells after 100 nM of tolytoxin treatment (Fig. 2b, red arrows). Subsequently, the effect of tolytoxin treatments on microtubule and actin cytoskeleton was evaluated in SW13 and SH-SY5Y cells. Briefly, cells treated with 100 nM of tolytoxin for 18 h were labeled with rhodamine-phalloidin and beta-tubulin to visualize the actin and microtubule cytoskeleton, respectively. The actin and microtubule cytoskeletons were unaffected in Control and Me-control, but cells treated with tolytoxin exhibited disrupted actin cytoskeleton, which was detected as disappearance of intact actin filaments (Fig. 2c. arrows) and appearance of interrupted actin fibers which were detected as punctate labeling (Fig. 2c. arrowheads). Microtubule cytoskeleton remained unaffected under these conditions for both cell types. In addition, to evaluate the G-actin content in the presence of tolytoxin, both cell types were treated with 100 nM of tolytoxin and labeled for Pan-Actin and F-actin (immunocytochemistry with Pan-actin antibody and rhodamine phalloidin staining, respectively; Supplementary Fig. 2a). Pan-actin / F-actin ratio was calculated by measuring the total integrated density of both labelling per cell. This ratio was significantly increased in both cell types, indicating that our tolytoxin induces actin depolymerization (Supplementary Fig. S2b), as shown before^29,33^. Overall, these results suggest that in SW13 and SH-SY5Y cells tolytoxin affects cell viability, cell proliferation and actin cytoskeleton, but not microtubules.

### Tolytoxin specifically decreases the number of morphologically defined TNTs but not attached filopodia in SW13 and SH-SY5Y cells

**Figure 3:**
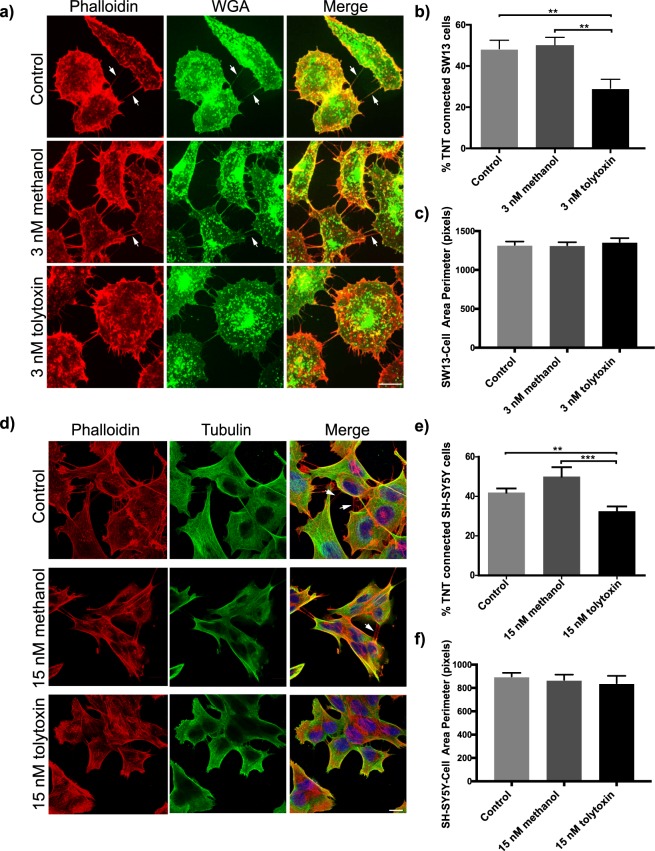
Tolytoxin specifically decreases the number of TNT connected cells (**a)** Rhodamine phalloidin (for actin) and WGA (for membrane) staining in untreated control cells and 3 nM methanol or 3 nM tolytoxin treated SW13 cells. Arrows are indicating the TNTs not touching to the substrate (note that a representative image was selected in a non-TNT containing area since such areas were more pronounced with tolytoxin treatment). Scale bar: 10 μm. (**b**) Graph showing the percentage of TNT connected cells in Control, 3 nM Me-control or 3 nM tolytoxin treated cells. Data were collected from three independent experiments, 60–80 cells were analysed per condition (p = 0.937 for Control *versus* 3 nM Me-control, p = 0.0038 for Control *versus* 3 nM tolytoxin, p = 0.0013 for 3 nM Me-control *versus* 3 nM tolytoxin. Mean percentage of TNT connected cells in Control: 48.37% ± 4.17, Me-control: 50.33% ± 3.60, 3 nM tolytoxin: 29.23% ± 4.37). (**c**) Measurement of SW13 cell perimeter in Control, Me-Control and 3 nM tolytoxin treated cells from three independent experiments (40 cells were analysed per condition). Mean perimeter in Control: 1324 ± 42, Me-control: 1318 ± 39, 15 nM tolytoxin 1360 ± 49 (p = 0.995 for control *versus* 3 nM Me-control, p = 0.827 for control *versus* 3 nM tolytoxin p = 0.769 for 3 nM Me-Control control *versus* 3 nM tolytoxin). (**d**) Actin and microtubule cytoskeleton labeling with rhodamin phalloidin and beta-tubulin respectively in parallel to nuclear DAPI labeling in Control, 15 nM methanol and 15 nM tolytoxin treated SH-SY5Y cells. Arrows indicate TNTs. Scale bar: 10 μm. (**e**) Graph showing the percentage of TNT connected cells in Control, 15 nM Me-control or 15 nM tolytoxin treated cells from three independent experiments, 60–80 cells were analysed per condition (p = 0.6702 for Control *versus* 15 nM Me-control, p = 0.0073 for Control *versus* 15 nM tolytoxin, p = 0.0009 for 15 nM Me-Control *versus* 15 nM tolytoxin). Mean percentage of TNT connected cells in Control: 43.16% ± 42.15, Me-control: 46.37% ± 2.82, 15 nM tolytoxin: 32.53% ± 2.49. (**f**) Measurement of cell perimeter in Control, Me-Control and 15 nM tolytoxin treated cells from three independent experiments (40 cells were analysed per condition). Mean perimeter in Control: 899.1 ± 30.42, Me-control: 869.5 ± 45.31, 15 nM tolytoxin: 841.2 ± 62.88; (p = 0.873 for control *versus* 15 nM Me-control, p = 0.676 for control *versus* 15 nM tolytoxin, p = 0.906 for 15 nM Me-control *versus* 15 nM tolytoxin).

Next, we analysed the effect of tolytoxin on TNTs. To this aim SW13 cells were treated with different concentrations of tolytoxin ranging from 0.5 nM to 20 nM, and morphologically defined TNTs (straight connections not touching to the substrate; illustrated in Supplementary Fig. 2c) were counted manually (Supplementary Table S1) following a protocol previously set up^36^. The minimal concentration of tolytoxin sufficient to significantly decrease the number of TNTs was detected as 3 nM (Fig. 3a). The percentage of TNT connected cells in the Control and Me-control were found to be 48.37% and 50.33%, respectively, whereas only 29.23% of the cells treated with 3 nM tolytoxin were connected by TNTs (p = 0.937 for control *versus* 3 nM Me-control, p = 0.0038 for control *versus* 3 nM tolytoxin, p = 0.0013 for 3 nM Me-control *versus* 3 nM tolytoxin; Fig. 3b).

To rule out that the effect of tolytoxin on TNTs was specific for SW13 cells we repeated the TNT counting experiments in neuronal SH-SY5Y cells. We chose this cell line for two main reasons: (i) Because of its different origin from epithelial cells and as they are human neuronal cell model for neurodegenerative diseases, (ii) Because the TNT structure in these cells has been thoroughly characterized by fluorescence and cyro-correlative light and electron microscopy (FM and cryo-CLEM) and by correlative focused ion beam scanning electron microscopy (FIB-SEM)^5^. We demonstrated that in SH-SY5Y cells, TNTs identified by FM (following the criteria described in materials and methods), are indeed structurally unique and different from filopodia (or from previously described cytonemes) and they are open at both ends, thus allowing the transfer of different cargoes^5^. Thus, we treated SH-SY5Y cells with 3 nM to 15 nM of tolytoxin (Supplementary Table S1). The percentage of TNT-connected cells significantly decreased at 15 nM of tolytoxin (p = 0.6702 for Control *versus* 15 nM Me-control, p = 0.0073 for Control *versus* 15 nM tolytoxin, p = 0.0009 for 15 nM Me-control *versus* 15 nM tolytoxin; Fig. 3d). In Control and Me-control conditions, the percentage of TNT-connected cells were found to be 43.16% and 46.37%, respectively, whereas it was 32.53% in the presence of 15 nM tolytoxin (Fig. 3e). Importantly, no effect on global actin and microtubule cytoskeleton was observed at these tolytoxin concentrations.

In addition, measurements of individual cell perimeters in all treatment conditions indicated that tolytoxin did not induce any significant change in both cell types (Fig. 3c,f). Mean perimeter of SW13 cells was detected as 1324, 1318 and 1360 pixels in Control, Me-Control and 3 nM tolytoxin treated cells, respectively (p = 0.999 for control *versus* 3 nM Me-control, p = 0.956 for control *versus* 3 nM tolytoxin, p = 0.929 for 3 nM Me-control *versus* 3 nM tolytoxin). Mean perimeter of SH-SY5Y cells was detected as 899.1, 869.5 and 841.2 pixels in Control, Me-Control and 15 nM tolytoxin treated cells, respectively (p = 0.750 for control *versus* 15 nM Me-control, p = 0.608 for control *versus* 15 nM tolytoxin, p = 0.975 for 15 nM Me-control *versus* 15 nM tolytoxin).

In order to understand whether the effect of the toxin was specific for TNTs, we decided to assess whether the same concentrations of tolytoxin had an effect on attached filopodia formation using an established assay to count attached filopodia^27,37–39^. To this aim SW13 and SH-SY5Y cells treated for 18 h with 3 or 15 nM of tolytoxin respectively, were labelled with vinculin antibody, which specifically labels attached filopodia and then processed for quantitative immunofluorescence analysis. Of interest, we did not observe any significant difference between tolytoxin treated SW13 and SH-SY5Y cells compared to Control and Me-Control (Supplementary Fig. S3a,c). In SW13 cells average number of attached filopodia was 88.32 in control, 78.73 in Me-Control, 86.94 in 3 nM tolytoxin treated cells (p = 0.201 for control *versus* 3 nM Me-control, p = 0.623 for control *versus* 3 nM tolytoxin, p = 0.960 for 3 nM Me-control *versus* 3 nM tolytoxin; Supplementary Fig. S3b). The average number of attached filopodia detected in SH-SY5Y cells were 43.41 in Control, 57.85 in Me-Control, 39.42 in 15 nM tolytoxin treated cells and (p = 0.209 for control *versus* 15 nM Me-control, p = 0.679 for control *versus* 15 nM tolytoxin, p = 0.934 for 15 nM Me-control *versus* 15 nM tolytoxin; Supplementary Fig. S3d).

Overall, these data indicate that tolytoxin specifically decreases the number of TNTs (or TNT- like structures) without affecting attached filopodia formation. The decrease in TNT formation is not due to the changes in cell proliferation or to general morphological changes induced by the drug. We also show that the concentration needed to decrease TNT formation is cell type-dependent, suggesting that neuronal cells are more resistant to tolytoxin.

### Tolytoxin decreases mitochondria transfer in SW13 and SH-SY5Y cells

**Figure 4:**
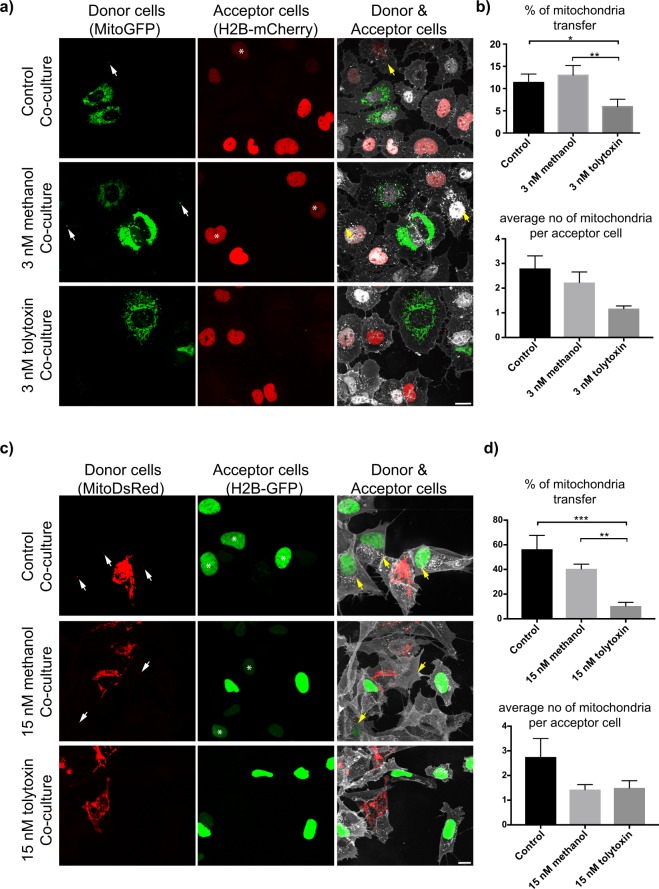
Tolytoxin decreases mitochondria transfer in SW13 and SH-SY5Y cells. (**a**) Mito-GFP transfected SW13 donor cells in Control, 3 nM Me-control and 3 nM tolytoxin co-cultures (left panel), acceptors transfected with H2B-mCherry (middle panel), donor and acceptor cells in different co-culture conditions (right panel). Arrows indicate mitochondria that had been received by acceptor cells marked with asterisk. Scale bar: 10 μm. (**b**) Upper graph showing the percentage of mitochondria transfer in each condition. Mean percentage of mitochondria transfer in Control: 11.34% ± 1.91, 3 nM Me-control: 12.99% ± 2.17, 3 nM tolytoxin: 4.84% ± 1.31 (p = 0.80 for Control *versus* 3 nM Me-control, p = 0.043 for Control *versus* 3 nM tolytoxin, p = 0.0093 for 3 nM Me-control *versus* 3 nM tolytoxin). Lower graph showing the average number of mitochondria puncta per acceptor cell in each condition. Mean number of mitochondria puncta in Control: 2.8 ± 0.5, 3 nM Me-control: 2.22 ± 0.42, 3 nM tolytoxin: 1.16 ± 0.11 (p = 0.62 for Control *versus* 3 nM Me-control, p = 0.19 for Control *versus* 3 nM tolytoxin, p = 0.48 for 3 nM Me-control *versus* 3 nM tolytoxin). (**c**) Mito-DsRed expressing SH-SY5Y donors in Control, 15 nM Me-control and 15 nM tolytoxin co-cultures (left panel), H2B-GFP expressing acceptor cells (middle panel), donor and acceptor cells in different co-culture conditions (right panel). Arrows indicate mitochondria that had been received by acceptor cells marked with asterisk. Scale bar represents 15 μm. (**d**) Upper graph showing the percentage of mitochondria transfer in each condition. Mean percentage of mitochondria transfer in Control: 56.35% ± 11.3, 15 nM Me-control: 40.51% ± 3.77, 15 nM tolytoxin: 10.37% ± 2.99 (p = 0.188 for Control *versus* 15 nM Me-control, p = 0.0004 for Control *versus* 15 nM tolytoxin, p = 0.0069 for 15 nM Me-control *versus* 15 nM tolytoxin). Lower graph showing the average number of mitochondria puncta per acceptor cells in each condition. Mean number of mitochondria puncta in control: 2.75 ± 0.75, 15 nM Me-control: 1.42 ± 0.2 15 nM tolytoxin: 1.5 ± 0.28 (p = 0.08 for Control *versus* 15 nM Me-control, p = 0.15 for Control *versus* 15 nM tolytoxin, p = 0.99 for 15 nM Me-control *versus* 15 nM tolytoxin). Data were collected from three independent experiments where 60–80 cells were analysed per condition for both cell types.

Although from our morphological criteria we could conclude that tolytoxin was affecting TNT-like structures, it is imperative to sustain morphological criteria with functional criteria when studying TNTs. Thus, next we evaluated whether the minimum concentrations of tolytoxin that decrease the number of TNTs would also impact the TNT-mediated transfer of vesicles between cells by performing co-culture experiments (Supplementary Fig. S4). First, SW13 donor cells were treated with 3 nM of tolytoxin prior to be labeled with DiD and mixed with H2B-mCherry-transfected acceptor cells (Supplementary Figs S4 and S5). After image acquisition, the percentage of DiD transfer and the number of DiD positive vesicles per acceptor cell were quantified. The percentage of DiD transfer was significantly higher in co-cultures having Control and Me-control (63% and 59% respectively, later referred as control co-cultures) than in co-culture having 3 nM tolytoxin treated donors (42%, here after tolytoxin co-culture; p = 0.777 for Control *versus* 3 nM Me-control, p = 0.0088 for Control *versus* 3 nM tolytoxin, p = 0.045 for 3 nM Me-control *versus* 3 nM tolytoxin; Supplementary Fig. S5a,b left graph). No difference in the average number of DiD-positive vesicles per acceptor cell was observed for any tested condition (Supplementary Fig. S5b right graph). In parallel, secretion test revealed 10% of DiD transfer was through secretion. These data suggest that 3 nM of tolytoxin is sufficient to decrease cell-to-cell contact mediated vesicle transfer between SW13 cells. DiD labeling targets all vesicle membranes regardless of their nature, however mitochondria were shown to be transferred at high rates, particularly between tumor cells to supply an energy source to recipient cells and to play a role in the maintenance and proliferation of cancer cells^17,26^. Thus, we next assessed the effect of tolytoxin on TNT- mediated mitochondria transfer. Co-culture experiments were performed both with SW13 and SH-SY5Y cells (Supplementary Fig. S4b,c). Mito-GFP transfected SW13 cells and SH-SY5Y cells stably expressing Mito-DsRed were used as donors and treated with 3 nM and 15 nM of tolytoxin, respectively (Fig. 4a,c, left column). H2B-mCherry transfected SW13 cells and SH-SY5Y cells stably expressing H2B-GFP were used as acceptors (Fig. 4a,c, middle column). Donor and acceptor cells were co-cultured for 24 h (Fig. 4a,c, right column). After image acquisition, the percentage of mitochondria transfer and the number of mitochondria received per acceptor SW13 cells were quantified for each condition. Mitochondria transfer in tolytoxin co-culture was significantly lower (4.84%) compared to control co-cultures (p = 0.8 for Control *versus* 3 nM Me-control, p = 0.04 for Control *versus* 3 nM tolytoxin, p = 0.009 for 3 nM Me-control *versus* 3 nM tolytoxin; Fig. 4b, upper panel). We did not observe any significant difference in the average number of mitochondria received per acceptor cells for these conditions (Fig. 4b, lower panel).

Similarly, for the SH-SY5Y cells, mitochondria transfer in co-cultures in the presence of 15 nM tolytoxin was significantly lower than in control co-cultures (10% *versus* 56% and 40% in control co-cultures, p = 0.188 for Control *versus* 15 nM Me-control, p = 0.0004 for Control *versus* 15 nM tolytoxin, p = 0.0069 for 15 nM Me-control *versus* 15 nM tolytoxin; Fig. 4d upper panel). The average number of mitochondria received per acceptor cells was not significantly different (Fig. 4d, lower panel, a representative orthogonal image of an acceptor cell received mitochondria is also presented in Supplementary Fig. 2e).

As control for cell-to-cell contact mediated transfer we analysed the occurrence of mitochondria transfer after challenging acceptor cells with the conditioned media from donor cells for 24 h (Supplementary Fig. S4e). No transfer of mitochondria from donor to acceptor cells (both in SW13 and SH-SY5Y) occurred through the conditioned media of donor cells, indicating that transfer does not occur though a secretory mechanism, but relay on cell to cell contact (a representative image of mitochondria inside of TNT is presented in control cells in Supplementary Fig. 2d, left panels).

Overall, these data suggest that 3 nM and 15 nM of tolytoxin significantly decreased the number of TNT-connected SW13 and SH-SY5Y cells, and TNT-mediated vesicle and mitochondria transfer.

### Tolytoxin decreases α-synuclein fibril transfer in SH-SY5Y cells

**Figure 5:**
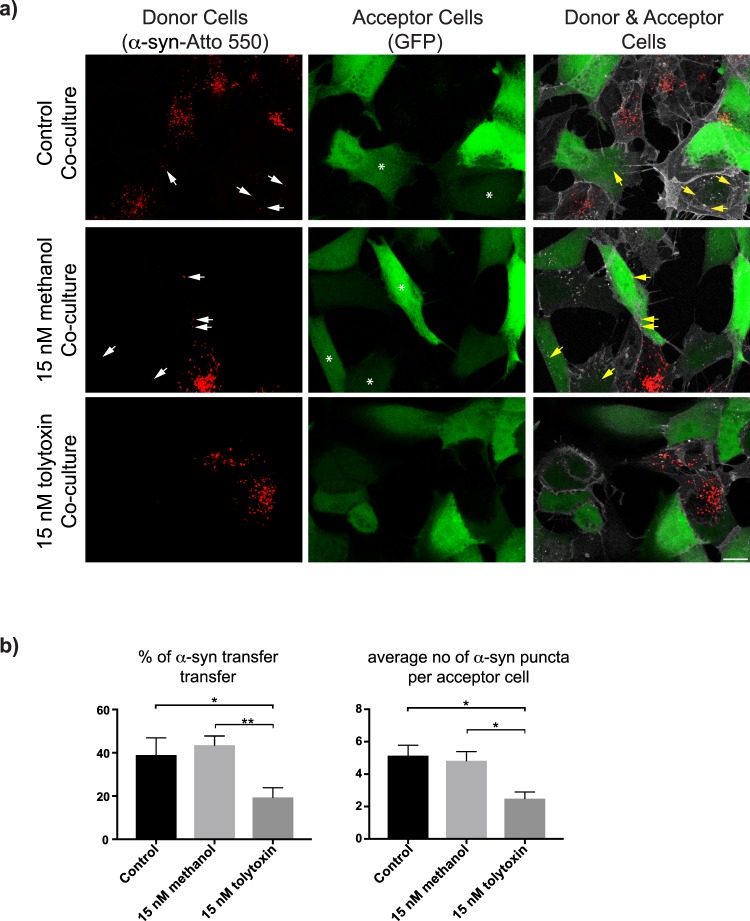
Tolytoxin decreases α-syn fibril transfer in SH-SY5Y cells. (**a**) α-syn loaded SH-SY5Y donor cells in Control, 15 nM Me-control and 15 nM tolytoxin co-cultures (left panel), GFP transfected acceptor cells (middle panel), donor and acceptor cells mixed with 1:1 ratio in different co-culture conditions (right panel). Arrows indicate α-syn fibrils that had been received by acceptor cells. Scale bar: 15 μm. (**b**) Left graph showing the percentage of α-syn transfer in each co-culture condition. Mean percentage of α-syn transfer in Control: 38.96% ± 7.89, 15 nM Me-control: 43.54% ± 4.23, 15 nM tolytoxin: 19.4% ± 4.44 (p = 0.829 for Control *versus* 15 nM Me-control, p = 0.04 for Control *versus* 15 nM tolytoxin, p = 0.0061 for 15 nM Me-control *versus* 15 nM tolytoxin. Right graph showing the average number of α-syn puncta per acceptor cell in each co-culture condition. Mean number of α-syn puncta in Control: 5.14 ± 0.63, 15 nM Me-control: 4.82 ± 0.57, 15 nM tolytoxin: 2.49 ± 0.4 (p = 0.90 for Control *versus* 15 nM Me-control, p = 0.01 for Control *versus* 15 nM tolytoxin, p = 0.02 for 15 nM Me-control *versus* 15 nM tolytoxin). Data were collected from four independent experiments where 60–80 cells were analysed per condition.

We previously showed that α-syn fibrils traffic between cells through TNTs^14^ leading to dissemination of misfolded protein in the culture. Thus, TNTs could be a suitable therapeutical target to stop the spreading of synucleopathies. We therefore investigated whether tolytoxin also impacted TNT-mediated α-syn fibril transfer (illustrated in Supplementary Fig. 2d right panels). Co-culture experiments were performed with donor SH-SY5Y cells, first challenged with ATTO-550-tagged α-syn fibrils and then treated with 15 nM of tolytoxin prior to 24 h of incubation with GFP transfected acceptor cells (Fig. 5a, Supplementary Fig. S4d). After image acquisition, transfer of α-syn fibrils and the average number of α-syn puncta received by acceptor cells were quantified. In 15 nM tolytoxin co-culture the percentage of transferred α-syn fibril (19.4%) was significantly lower than that in control co-cultures (38.96% and 43.54% in Control and Me-control respectively, p = 0.829 for Control *versus* 15 nM Me-control, p = 0.04 for Control *versus* 15 nM tolytoxin, p = 0.0061 for 15 nM Me-control *versus* 15 nM tolytoxin; Fig. 5b, left panel). The average number of α-syn puncta was 2.49 in 15 nM tolytoxin co-culture, which was also significantly lower compared to control co-cultures (p = 0.90 for Control *versus* 15 nM Me-control, p = 0.01 for Control *versus* 15 nM tolytoxin, p = 0.02 for 15 nM Me-control *versus* 15 nM tolytoxin; Fig. 5b, right panel). Only 2% of α-syn were detected to be transferred by secretion. These results suggest that 15 nM of pure tolytoxin efficiently decreases α-syn fibril transfer and the number of α-syn puncta received by acceptor cells.

### Pure tolytoxin from *Scytonema* sp. PCC 10023 recapitulates the effect of tolytoxin from *Planktothrix paucivesiculata* on TNTs

**Figure 6:**
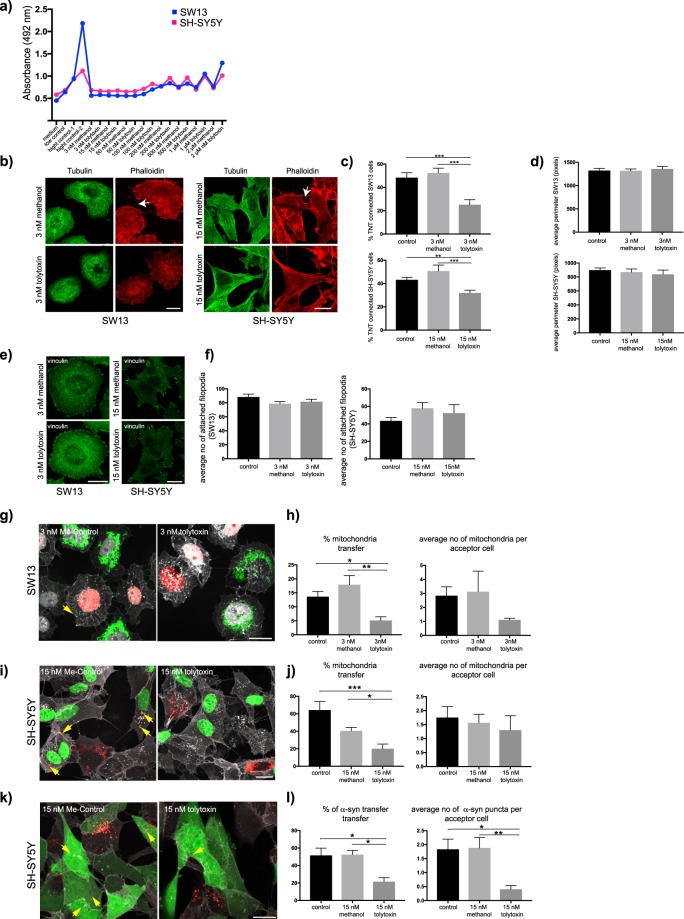
Effect of pure tolytoxin from *Scytonema* sp. PCC on SW13 and SH-SY5Y cells (**a**) Graph showing average LDH release for SW13 and SH-SY5Y cells. Data were collected from two independent experiments. Medium: negative control, low control: LDH activity released from the untreated cells, high control-1 and 2: cells lysed with 5 and 10 μl of lysis buffer respectively. (**b**) Representative image of actin and microtubule labeling. Arrows indicate TNTs, Scale bar: 10 µm. (**c**) Upper graph: mean percentage of TNT connected SW13 cells, lower graph: percentage of TNT connected SH-SY5Y cells from three independent experiments (50–60 cells were analysed per condition). (**d**) Upper graph: average perimeter of SW13 cells in Control: 1324 ± 42, Me-control: 1318 ± 39, 3 nM tolytoxin: 1357 ± 53 (p = 0.995 for control *versus* 3 nM Me-control, p = 0.86 for control *versus* 3 nM tolytoxin p = 0.8 for 3 nM Me-Control control *versus* 3 nM tolytoxin). Lower graph: average perimeter of SH-SY5Y cells in Control: 899.1 ± 30.42, Me-control: 869.5 ± 45.31, 15 nM tolytoxin 838.9 ± 60.35; (p = 0.862 for control *versus* 15 nM Me-control, p = 0.662 for control *versus* 15 nM tolytoxin, p = 0.894 for 15 nM Me-control *versus* 15 nM tolytoxin). Data were collected from three independent experiments; 40 cells were analysed per condition. (**e**) Vinculin labeling in SW13 and SH-SY5Y cells. (**f**) Left graph: average number of attached filopodia in SW13 cells. Right graph: average number of attached filopodia in SH-SY5Y cells. Data were collected from three independent experiments; 40 cells were analysed per condition. (**g)** Mitochondria transfer in SW13 cells, (**h)** Left graph: the percentage of transfer, Right graph: average number of mitochondria in acceptor cells, Control: 2.83 ± 0.63, Me-control: 3.12 ± 1.46, 3 nM tolytoxin: 1.11 ± 0.11 (p = 0.96 for Control *versus* 3 nM Me-control, p = 0.3 for Control *versus* 3 nM tolytoxin, p = 0.26 for 3 nM Me-Control *versus* 3 nM tolytoxin). (**i**) Mitochondria transfer in SH-SY5Y cells. (**j**) Left graph: percentage of transfer. Right graph: average number of mitochondria in acceptor cells, Control: 1.75 ± 0.39, Me-Control: 1.56 ± 0.3, 15 nM tolytoxin: 1.30 ± 0.51 (p = 0.94 for Control *versus* 15 nM Me-control, p = 0.74 for Control *versus* 15 nM tolytoxin, p = 0.89 for 15 nM Me-control *versus* 15 nM tolytoxin). (**k**) α-syn transfer in SH-SY5Y cells. (**l**) Left graph: percentage of transfer. Right graph: average number of α-syn in acceptor cells. Data for transfer experiments (mitochondria and α-syn) were collected from three independent experiments; 50–60 cells were analysed per condition.

To verify whether tolytoxin produced by another genus of cyanobacteria could induce similar effects, the same experiments were performed on pure tolytoxin extracted from *Scytonema* sp. PCC 10023 (10023 thereafter). First, the effect of tolytoxin 10023 on cell viability was tested by LDH release assay in wide range of concentrations (from 3 nM to 2 µM). Similar to 8926, LDH release was started to increase in the cell medium starting from 100 nM (Fig. 6a). Then, to test whether the minimal concentrations of tolytoxin 8926 (3 and 15 nM for SW13 and SH-SY5Y cells, respectively) would induce similar effect with tolytoxin 10023, we applied the same treatment for TNT counting experiments. Cells were also labelled for microtubule cytoskeleton in addition to the actin labelling to evaluate the effect of tolytoxin 10023 on both actin and tubulin cytoskeleton (Fig. 6b). Percentage of TNT connected SW13 cells were detected as 48.37% ± 4.16 and 52.35% ± 4.12 in Control and Me-Control conditions respectively whereas it was 25.14% ± 4.38 in 3 nM tolytoxin treated cells (p = 0.817 for control *versus* 3 nM Me-control, p = 0.0005 for control *versus* 3 nM tolytoxin, p = 0.0005 for 3 nM Me-control *versus* 3 nM tolytoxin; Fig. 6c, upper graph). This percentage was 43.16% ± 2.14 in Control, 50.7% ± 5.14 in Me-Control and 31.9% ± 2.38 in 15 nM tolytoxin treated SH-SY5Y cells (p = 0.24 for Control *versus* 15 nM Me-control, p = 0.0064 for Control *versus* 15 nM tolytoxin, p = 0.0005 for 15 nM Me-control *versus* 15 nM tolytoxin; Fig. 6c, lower graph). Again, no effect on microtubule cytoskeleton was detected. The average perimeter of the cells did not change for both cell types suggesting that tolytoxin do not impact the cell morphology (Fig. 6d). Next, we addressed attached filopodia formation in the same condition that for 8926. We did not observe any change in attached filopodia formation in the presence of tolytoxin 10023 for both cell types (Fig. 6e,f). In SW13 cells, average number of attached filopodia were detected as 88.32 ± 4.16, 78.73 ± 3.17 and 81.56 ± 3.45 in Control, Me-Control and 3 nM tolytoxin treated cells respectively. (p = 0.135 for control *versus* 3 nM Me-control, p = 0.470 for control *versus* 3 nM tolytoxin, p = 0.879 for 3 nM Me-control *versus* 3 nM tolytoxin; Fig. 6f, left graph). In SH-SY5Y cells average number of attached filopodia were detected as 43.41 ± 3.89, 57.85 ± 6.66 and 52.4 ± 9.64 in Control, Me-Control and 15 nM tolytoxin treated cells, respectively (p = 0.172 for control *versus* 15 nM Me-control, p = 0.559 for control *versus* 15 nM tolytoxin, p = 0.848 for 15 nM Me-control *versus* 15 nM tolytoxin; Fig. 6f, right graph).

Then, we addressed the cargo transfer in the presence of tolytoxin. Mitochondria transfer in 3 nM tolytoxin 10023 treated SW13 cells was significantly lower compared to control co-cultures (Fig. 6g). Percentage of transfer was detected as 13.63% ± 1.85 and 17.92% ± 3.28 in Control and Me-Control co-cultures respectively. In the presence of 3 nM tolytoxin, this percentage was significantly decreased and detected as 5.14% ± 1.37 (p = 0.38 for control *versus* 3 nM Me-control, p = 0.038 for control *versus* 3 nM tolytoxin, p = 0.004 for 3 nM Me-control *versus* 3 nM tolytoxin; Fig. 6h, left graph), whereas no change was observed in the average number of mitochondria transferred (Fig. 6h, right graph). Similarly, in SH-SY5Y cells 15 nM tolytoxin 10023 treatment significantly decreased the mitochondria transfer (Fig. 6i). Percentage of mitochondria transfer was detected as 64.29% ± 9.8 and 40.5 ± 3.71 in Control and Me-Control respectively whereas it was 20.08% ± 5.37 in 15 nM tolytoxin treated cells (p = 0.053 for control *versus* 15 nM Me-control, p = 0.0002 for control *versus* 15 nM tolytoxin, p = 0.04 for 15 nM Me-control *versus* 15 nM tolytoxin; Fig. 6j, left graph). The average number of mitochondria transferred from donor to acceptor cells remain unchanged (Fig. 6j right graph). Finally, the efficiency of α-syn fibril transfer in the presence of tolytoxin 10023 was addressed by performing co-culture experiments (Fig. 6k). Both transfer efficiency of fibrils and average number of α-syn puncta detected in acceptor cells were significantly decreased in the presence of 15 nM tolytoxin 10023 in SH-SY5Y cells. Percentage of transfer was detected as 51.56% ± 8.28 and 52.45% ± 4.73 in Control and Me-Control co-cultures, whereas it was significantly decreased and detected as 21.58% ± 4.45 in 15 nM tolytoxin treated co-culture (p = 0.99 for control *versus* 15 nM Me-control, p = 0.02 for control *versus* 15 nM tolytoxin, p = 0.03 for 15 nM Me-control *versus* 15 nM tolytoxin; Fig. 6l, left). The average number of α-syn fibril puncta in acceptor cells were detected as 1.83 ± 0.36 in Control, 1.88 ± 0.37 in Me-Control and 0.4 ± 0.13 in 15 nM tolytoxin treated co-cultures (p = 0.99 for control *versus* 15 nM Me-control, p = 0.01 for control *versus* 15 nM tolytoxin, p = 0.008 for 15 nM Me-control *versus* 15 nM tolytoxin, Fig. 6l, right).

Overall these results suggest that regardless of the producer, pure tolytoxin is able to significantly decrease the number of TNT connected cells and impair their transfer function, without affecting attached filopodia formation and without inducing significant morphological changes or cell death. We therefore argue that it can be use as specific drug to inhibit/destroy TNTs.

## Discussion

TNTs are actin-containing cytoplasmic bridges between cells that have emerged as a novel communication route^1^. They allow transfer of a wide range of cargoes including organelles and protein aggregates such as α-syn and prion. Their involvement in neurodegenerative diseases and cancer development makes them attractive therapeutic targets. Several reports demonstrated a significant increase in the number of TNTs in pathological conditions^14,20,23,24^ thus decreasing or blocking TNT formation in a specific manner is becoming a promising therapeutic approach. In this context, we used tolytoxin, a cyanobacterial macrolide known to inhibit actin polymerization extracted to high degree of purity from two different genera of cyanobacteria; *Planktothrix* and *Scytonema*, to study their effects on TNT formation and function in both neuronal and non-neuronal cells.

Previous studies reported that tolytoxin extracted from *Scytonema* strains is extremely toxic to human epidermoid carcinoma (KB) cells^29,40^. Even nM concentrations were shown to induce profound morphological changes beginning with the formation of zeiotic processes and culminating in nuclear protrusion, eventually inducing cell death. In rat smooth muscle cells (A-10), similar concentrations were shown to induce total actin cytoskeleton rupture^29,40^. Similarly it has been reported that tolytoxin is able to kill human breast carcinoma cells (MCF-7)^32^ and human ovarian carcinoma cells (SKOV-3)^33^ and proposed as a potential anti-cancer drug. Although we were able to recapitulate previously reported effects of tolytoxin (on cell viability, proliferation and morphology) on our cell models, the concentrations of tolytoxin needed to observe these effects were much higher. LDH release and cell proliferation were affected in SW13 and SH-SY5Y cells with at least 100 nM of pure tolytoxin. In addition, this tolytoxin concentration induced polynucleation in SW13 cells. Because we tested tolytoxin extracts in two cell types of different origin (human epithelial and human neuronal) and from two different genera of cyanobacteria, and revealed their high grade of purities by spectrum analyses, one possibility is that the discrepancy in the effect of tolytoxins could be due to a lower grade of purity of the tolytoxin used in previous studies^29,40^. In our case, tolytoxin is not associated with congeners and to other side-products or colloidal aggregates, which were shown to be the most common artifact in early drug discovery^41^. Alternatively, this discrepancy could be linked to the cell type used in the previous study. Indeed, in agreement with previous data^33^, we have observed a clear actin disruption in SKOV-3 cells treated with 3 and 15 nM of pure tolytoxin extracted from *Planktothrix paucivesiculata* (Supplementary Fig. 2f), whereas these concentrations did not induce any global actin disruption effect in SW13 and SH-SY5Y cells. This data suggests that SKOV-3 cells are more sensitive to tolytoxin compared to SW13 and SH-SY5Y cells. Thus, it will be interesting to further investigate the action of tolytoxin on a wide panel of different cells and assess whether a specific effect on TNTs is reproduced at different cell-specific concentrations.

Here we demonstrated that in SW13 and SH-SY5Y cells, nM concentrations of pure tolytoxin extracted from different producers are sufficient to significantly decrease the number of TNT-connected cells and transfer of different cargoes such as mitochondria and α-syn (as illustrated in Supplementary Fig. S2d), which has been shown to be transferred through TNTs^13,14,17,42^. Furthermore, we have recently demonstrated that the TNTs observed by FM in SH-SY5Y cells have a unique structure and are open ended^5^. Together with the effect on the transfer, we report here we are confident that tolytoxin acts on bona-fide TNT structures in SH-SY5Y cells. Although we cannot completely rule out that other actin based cellular structures could be affected by tolytoxin, this effect appeared to be specific for TNTs as in our hands it did not affect attached filopodia, another actin-based protrusions present in both cell types, and did not induce cell death or dramatic morphological changes that would in turn affect TNT formation.

Other compounds inhibiting actin polymerization such as cytochalasin B and D as well as latrunculin A were also shown to reduce TNT formation in various cell types, but mostly at µM concentrations^1,9,11,22,28^. To our knowledge the lowest concentration reported so far is 350 nM of cytochalasin B shown to decrease both TNT and filopodia formation^25^. Thus, compared to other actin depolymerizing agents, tolytoxin appears to be a very promising candidate for therapeutic approaches given the possibility of performing *in-vivo* studies considering its minimal effective concentrations.

We showed that the minimum effective concentration of pure tolytoxin needed to decrease TNT-like formation and cargo transfer was 3 nM for the epithelial SW13 cells, whereas it was five times higher for neuronal SH-SY5Y cells. These data revealed that TNTs of neuronal cells are more resistant to tolytoxin than the ones of non-neuronal cells and further suggested a cell type-dependent effect. TNT-like structures observed by FM were shown to be heterogeneous structures having different diameters and lengths in different cell types^43^. In addition, in some cell types microtubules were also detected in TNTs^9^. Thus, the difference in the minimal dose of tolytoxin required could be linked to the structural differences in TNTs. It would be therefore interesting to apply our cryo-CLEM and FIB-SEM approach to SW13 cells to reveal if the structure of TNT-like protrusion observed by FM in these cells is the same as we have shown for neuronal SH-SY5Y and CAD cells^5^. Furthermore, the molecular mechanism of how tolytoxin decreases TNT formation, and whether it also interacts with F-actin nucleating proteins remain to be elucidated.

In early publications, tolytoxin and its scytophycin congeners were considered as antifungal compounds^44^. While several *Planktothrix* populations were investigated for their resistance toward the parasitism of chytrids^45,46^, the tolytoxin pathway was only found in diverse strains of the benthic *Planktothrix* (PCC 11201^34^ and PCC 8926). This pathway also seems to have evolved toward a more elaborated pathway in *Planktothrix paucivesiculata* PCC 9631 allowing it to produce luminaolide B, a dimeric tolytoxin variant^35^. Along with the recent description of another antifungal compound, hassallidin E, in *Planktothrix serta* PCC 8927^47^, the few genomes of benthic *Planktothrix* studied seems to possess different antifungal compounds in comparison to any other genomes of planktic *Planktothrix*. Whether the benthic *Planktothrix* are more prone to be infected by chytrids than the planktic ones remains to be investigated.

In conclusion, our results show that nM concentrations of pure tolytoxin from different cyanobacterial genera are sufficient to specifically decrease the number of functional TNTs and to affect the transfer of mitochondria and α-syn fibrils, which have been proposed to have a relevant role in the development and progression of cancer and Parkinson’s Disease respectively. Importantly, we show that the effect is specific for TNTs and does not cause a global collapse in actin cytoskeleton or induce cell death. To our knowledge, this study presents for the first time the effect of tolytoxin on TNT formation and function. Thus, we propose that tolytoxin could be a novel candidate for therapeutic approaches against neurodegenerative diseases and cancer.

## Methods

### Cyanobacterial production of the tolytoxin

A tolytoxin like *trans*-AT PKS cluster was detected during ongoing genome sequencing of *Planktothrix paucivesiculata* PCC 8926^48^. The gene cluster was compared to the tolytoxin and luminaolide B biosynthetic gene clusters previously described^34,35^. Genomic comparison showed these clusters highly similar to the one present in *Planktothrix* sp. PCC 11201, although the strain was more closely related to the *P. paucivesiculata* PCC 9631 producing luminaolide B (Supplementary Fig. S1). Isolation and characterization of tolytoxin in the strain PCC 8926 was performed by HPLC-MS and NMR as described for the characterization of this compound in *Scytonema* sp. PCC 10023^35^. NMR spectra were recorded on a Bruker Avance III spectrometer equipped with a cold probe at 500 MHz for ^1^ H NMR. Chemical shifts were referenced to the solvent peaks at δ_H_ 2.05 for acetone-*d*_6_. LC-ESI mass spectrometry was performed on a Thermo Scientific Q Exactive mass spectrometer. Pure tolytoxin was collected from peak eluted between 11.5–12.5 min on HPLC (UV 260 nm, Phenyl-hexyl column, 65% MeCN), and kept dry until experimental use. A tolytoxin stock solution of 0.7 mg/ml was prepared by dissolving pure tolytoxin in 90% methanol. Methanol as a control was used at the same concentration of tolytoxin in all the experiments. Cell treatments were performed via the medium with the appropriate concentration of tolytoxin diluted in PBS. Cells were incubated with tolytoxin at 37 °C for 18 h in all experimental procedures.

### Cell culture and transfection

SW13 cells were cultured in DMEM-1x (Gibco) having 10% foetal bovine serum (FBS), 1% penicillin-streptomycin (Pen-Strep), 1% non-essential amino acids (Gibco), 0.1% Glutamax (Gibco), and 1% Sodium Puryvate (Gibco). SH-SY5Y cells were cultured in RPMI (Euroclone) having 10% FBS and 1% Pen-Strep. SKOV-3 cells were cultured in RPMI medium including 1% Glutamax and 10% FBS. SW13 cells were transiently transfected with Mito-GFP, GFP and H2B-mCherry plasmids by lipofectamine 2000 (Invitrogen) according to the manufacturer’s instructions.

Stable cell line generation: 600.000 SH-SY5Y cells were plated on 60 mm plates and transfected with 0.5 µg of H2B-GFP plasmid. After 48 h, cells were transferred into 100 mm plates and cultured in RPMI complete medium including G418/ Geneticin. 15 days later, positive clones were selected.

Transduction of SH-SY5Y cells with a lentiviral vector expressing pCMV-Mito-DsRed: 600.000 SH-SY5Y cells were plated in 60 mm plates. After 24 h, they were infected with 800 µl of LV-CMV-Mito-DsRed. After 48 h, cells expressing Mito-DsRed have been validated.

### Cell viability assay

SW13 cells were treated with different concentrations of tolytoxin, and cell viability was measured by lactate dehydrogenase (LDH) release into the medium. Cytotoxicity Detection Kit^PLUS^ (Roche) was used to evaluate LDH release by following manufacturer’s instructions.

### Live cell analysis by Incucyte ZOOM

SW13 (50.000 cells/well) and SH-SY5Y (100.000 cells/well) were plated on B12 well plates (Falcon). After 24 h, cells were treated with desired concentration of tolytoxin and immediately transferred to incubator of Incucyte where automatic imaging of cells from nine different positions per well were acquired on live cells in every 30 min during 60 h. Data were then analyzed for percentage of confluency by using Incucyte ZOOM software.

### Microtubule, F and G actin and membrane detection

SW13 (75.000 cells per 35 mm Ibidi μ-dishes) and SH-SY5Y (100.000 cells per 12 mm glass coverslips) were plated. After 24 h, the cells were treated with desired concentration of tolytoxin, methanol in parallel to non-treated control cells. After 18 h of treatment, cells were washed with pre-warmed PHEM buffer (60 mM PIPES pH 6.9, 25 mM Hepes, 10 mM EGTA, 2 mM MgCl2 in H_2_O) prior to be fixed with 4% paraformaldehyde (PFA) and 0.05% glutaraldehyde (GA) in PHEM for 20 min at 37 °C. For microtubule detection, cells were then incubated in a 50 mM NH_4_Cl solution for 15 min at room temperature (RT) in order to quench PFA and GA autofluorescence. Cells were further permeabilized with 0.1% Triton X-100 in PBS for 2 min (for Pan-actin labeling 0.05% triton was used in this step). After several washes with PBS, aspecific antibody binding sites were blocked by using 2% bovine serum albumin (BSA) in PBS for 30 min. Cells were then incubated for 1 h with mouse anti-alpha tubulin antibody (T9026, Sigma) diluted 1:500 in blocking solution for microtubule detection or with mouse anti actin monoclonal C4 antibody (MP biomedicals, 1:200) for Pan-actin detection. Further washed cells were incubated with goat anti-mouse Alexa Fluor©-488 nm (Invitrogen) diluted 1:500 in blocking solution for 40 min. For F-actin detection, cells were stained with 0.6 μM rhodamine-phalloidin in PBS for 20 min. In subset of experiments, rhodamine-phalloidin staining was performed right after the fixation and cells were further stained by 3.3 μg.μL-1 Wheat Germ Agglutinin (WGA) Alexa Fluor©-488 nm conjugate solution (Invitrogen) in PBS for 20 min at RT for membrane detection. Finally, cells were washed and mounted with Aqua-Poly/Mount (Polysciences, Inc.), which has been systematically used in all experiments. To assess the changes in F and G-actin, control and tolytoxin treated cells labelled with anti actin clone C4 antibody and rhodamine phalloidin were imaged by confocal microscopy where all the settings kept the same. Quantification was performed by using ImageJ software; first a ROI delimiting each cell were created by free-hand drawing tool then total fluorescent intensity per cell were measured for each labeling. Data were collected in AU and presented as Pan-Actin/F-actin ratio.

### Attached filopodia detection

SW13 (75.000 cells per 35 mm Ibidi μ-dishes) and SH-SY5Y (100.000 cells per 12 mm glass coverslips) were plated. After 24 h, the cells were treated with desired concentration of tolytoxin or methanol in parallel to non-treated control cells. Cells were then fixed with 4% PFA for 20 min at RT and washed three times with PBS1X. Cells were then incubated for permeabilization and blocking with 2% BSA including 0.0075% saponin at RT for 1 h. Primary monoclonal antibody of vinculin (Sigma, 1:500) was prepared in PBS having 2% BSA and 0.01% saponin and incubated at RT for 1 h. After several washes with PBS 1X cells were incubated with secondary antibody goat anti-mouse Alexa Fluor©-488 nm (Invitrogen) in the same solution at RT for 1 h. Cells were then stained with rhodamine phalloidin (for SH-SY5Y cells) or with HCS Cell Mask^TM^ Blue Stain (Invitrogen, 1:5000) in PBS1X for 20 min. After several washes cells were labeled with DAPI in PBS1X at RT for 5 min and mounted. Quantification was performed as described before^27,37–39^ by (i) creating the ROI restricted to the outer region of the cells that covers only attached filopodia; (ii) automatized counting of the vinculin positive filopodia using spot detector tool (ICY software).

### TNT counting

Whole volume of actin labeled SW13 and SH-SY5Y cells were imaged by confocal microscopy (Zeiss-LSM700) controlled by ZEN software with 0.2 μm and 0.5 μm Z-stacks respectively. After image acquisition, remote cells connected by TNTs were manually counted by ICY software using semi-automatized TNT counting tool as previously described^36^. TNTs were discriminated from other protrusions as follows: all types of connections between each cell-pair were evaluated by scanning through the Z-stacks and connections that are fitting to the criteria of (i) not touching to the substratum, thus projections present in upper stacks of the image (Supplementary Fig. S2c); (ii) thinner than 500 nm; (iii) projections clearly starts from one cell and uninterruptedly continue towards the other cell to form a “bridge” were counted as TNTs. By using the semi-automatized TNT counting tool, total number of cells were counted by simply clicking on each cell and the cells connected by TNTs (fulfilling the criteria defined above) were connected manually by drawing a line using a free-hand line tool and counted automatically by the software. Thus, data were presented as percentage of TNT connected cells (TNT connected cells over total number of cells). TNT counting experiments has been coupled to all the co-culture experiments performed in this study since material transfer is also an important criterion of TNTs which distinguish them from other types of protrusions such as filopodia that does not allow material transfer between cells^5,27,49–51^.

### Preparation of α-syn fibrils

For fibril formation, recombinant full-length, wild-type human α-syn, purified as described previously^52^, was incubated in buffer A (50 mM Tris–HCl, pH 7.5, 150 mM KCl) at 37 °C under continuous shaking in an Eppendorf Thermomixer set at 600 rpm^53^. Fibrillar α-syn was centrifuged twice at 15.000 *g* for 10 min, suspended twice in PBS, and labeled with ATTO-550 (ATTO-Tec GmbH) NHS fluorophore following the manufacturer’s instructions using a protein/dye ratio of 1:2 as previously shown^43^. ATTO-550 α-syn fibrils were fragmented at 30 °C for 15 min in 2-mL Eppendorf tubes in a VialTweeter powered by an ultrasonic processor UIS250v (250 W, 24 kHz, Hielscher Ultrasonic, Teltow, Germany) set at 75% amplitude, 0.5 s pulses every 1 s. The nature of fibrillar α-syn forms before and after fragmentation was assessed using a JEOL 1400 transmission electron microscope following adsorption onto carbon-coated 200-mesh grids and negative staining with 1% uranyl acetate.

### Co-Culture preparation for mitochondria and α-syn fibril transfer experiments

Donor cells (either stably expressing Mito-Dsred or transfected with Mito-GFP or loaded with α-syn fibrils) were mixed in a ratio of 1:1 with the acceptor cells (either stably expressing fluorescently tagged human histone *H2B* gene or transfected with H2B-GFP or GFP plasmids) and co-cultured for 24 h prior to quantification. The percentage of mitochondria and α-syn fibril transfer from donor to acceptor cells and the average number of mitochondria and α-syn puncta per acceptor cell were quantified for both cell types. The details of the co-culture experiments are indicated in the supplementary information.

### Statistical analysis

One-way ANOVA and multiple comparison tests were used to evaluate the significance of TNT counting, DiD positive vesicle, mitochondria and α-syn transfer quantifications. Unpaired t-test was applied to comparisons of two conditions presented in Fig. 6. The bar graphs presented in the figures are indicating the mean values ± standard error of the mean. All column graphs and statistical analysis were performed by using GraphPad Prism version 7 software.

## Supplementary information

Supplementary Info

## Data Availability

The datasets generated during and/or analysed during the current study are available from the corresponding authors on reasonable request.
