# Supplementary material for: Effect of tolytoxin on tunneling nanotube formation and function
Source: Sci Rep. 2019 Apr 5;9:5741. doi: 10.1038/s41598-019-42161-6 (PMC6450976; doi:10.1038/s41598-019-42161-6)
Supplement: Supplementary file 1 — Supplementary Info [file 41598_2019_42161_MOESM1_ESM.pdf]

# Effect of tolytoxin on tunneling nanotube formation and function

Aysegul Dilsizoglu Senol[1], Anna Pepe[1], Clara Grudina[1], Nathalie Sassoon[2], Ueoka Reiko[3], Luc Bousset[4], Ronald Melki[4], Jörn Piel[3], Muriel Gugger[2*], Chiara Zurzolo[1*]

[1]Institut Pasteur, Unité Trafic Membranaire et Pathogenèse, 25–28 Rue du Docteur Roux, 75724 Paris CEDEX 15, France. [2]Institut Pasteur, Collection des Cyanobactéries, 25–28 Rue du Docteur Roux, 75724 Paris CEDEX 15, France.[3]Institute of Microbiology, Eigenössische Technische Hochschule (ETH) Zurich, Vladimir-Prelog-Weg 4, 8093 Zurich, Switzerland. [4]Paris-Saclay Institute of Neuroscience, CNRS, 1 Avenue de la Terrasse, 91190 Gif-sur-Yvette, France. *Correspondence and requests for materials should be addressed to MG (muriel.gugger@pasteur.fr) or CZ (chiara.zurzolo@pasteur.fr).

## Supplementary material

## Methods

### Co-Culture Experiments

*Co-culture preparation for DiD vesicle transfer experiments*

SW13 cells (donor) were plated on 35 mm dishes (Falcon) and treated with 3 nM tolytoxin or 3 nM methanol for 18 h in parallel to non-treated control cells. Then they were incubated with vybrant DiD cell labeling solution (Thermo Fisher Scientific) (1:2000 in complete medium) for 45 min at 37 °C to label vesicle membranes. Cells were washed three times with 0.01% trypsin to remove unbound dye. In parallel, SW13 cells (acceptor) were plated on T25 flasks and transfected with 3 μg of H2B-mCherry plasmid. Donor and acceptor cells were trypsinized, centrifuged (1000 rpm, 4 min), counted and co-cultured on 35 mm Ibidi μ-dishes for 24 h with 1:1 ratio (120.000 donor-120.000 acceptor). Same ratio was used for all co-culture preparations in SW13 cells (Supplementary Fig. S4a). Cells were then fixed with 4% of PFA for 20 min at RT. After several washes with PBS, cells were stained with HCS Cell Mask™ Blue Stain (Invitrogen, 1:5000) in PBS1X for 20 min and DAPI (1:5000) at RT for 5 min and then mounted. Image stacks (0.5 μm) covering the whole cellular volume were acquired using a confocal-microscope (Zeiss LSM700) with a 63X objective controlled by ZEN software for all co-culture experiments. Images were quantified for (i) the number of acceptor cells which had received the DiD labeled vesicles and (ii) the number of DiD labeled vesicles per acceptor cell.

Acceptor cells having DiD vesicles were detected by a script written for detection of cells having two colors (i.e. red and far red for acceptor cells and DiD labeled vesicles respectively). Number of DiD vesicles per acceptor cell were automatically counted by spot detector integrated into the same script. This script produced in ICY software was applied to all co-culture experiments by adjusting the color settings.

*Secretion test*

To evaluate the possibility of DiD labeled vesicle transfer from donor to acceptor cells mediated by secretion, donor cells were loaded with DiD, washed with 0.01% trypsin, cultivated into fresh medium and incubated for 24 h. Conditioned medium collected from these cells was centrifuged at 1000 rpm for 4 min to remove floating cells and added on acceptor cells. After 24 h, cells were fixed with 4% PFA at RT for 20 min. After image acquisition, acceptor cells were counted for the presence of DiD positive vesicles. Secretion test was performed in parallel to all the co-coculture experiments performed in this study by following the same protocol (Supplementary Fig. S4e).

*Co-culture preparation for mitochondria transfer experiments*

For SW13 cells, donor population were plated on T25 flasks (800.000 cells) and transfected with 3 μg of Mito-GFP for 24 h. Transfected cells were plated on 35 mm dishes and treated with 3 nM tolytoxin or 3 nM methanol for 18 h or left untreated. Meanwhile, acceptor cells were plated on T25 flasks and transfected with 3 μg H2B-mCherry plasmid. For SH-SY5Y cells, stable cell line expressing Mito-DsRed was used as donor population and cells were directly plated on 35 mm dishes and treated with 15 nM tolytoxin or 15 nM methanol for 18h or left untreated. As acceptor cell population, stable cell line expressing H2B-GFP was used. Donor and acceptor cells were mixed in 1:1 ratio (same concentration used in DiD transfer was applied to SW13 cells and 50.000 donors and 50.000 acceptor SH-SY5Y cells were plated on 12 mm glass coverslips) and co-cultured for 24 h (Supplementary Figs. S4b, c). After fixation with 4% PFA min at RT for 20, cells were stained for WGA Alexa Fluor©-647 nm (1:300) conjugate and DAPI prior to mounting. After image acquisition, number of acceptor cells, which had received mitochondria and the number of mitochondria received per acceptor cell were quantified. In parallel, secretion of mitochondria was also tested. Briefly, supernatant collected from the conditioned medium of SW13 donor cells (Mito-GFP transfected) and SH-SY5Y donor cells (Mito-DsRed expressing cells) were added onto acceptor cell populations (H2B-mCherry transfected SW13 cells or H2B-GFP expressing SH-SY5Y cells). Acceptor cells were then analysed for the presence of Mito-GFP.

*Co-culture preparation for* α-syn *fibril transfer experiments*

1 µM of ATTO-550 tagged α-syn fibrils were diluted in SH-SY5Y cell culture medium and sonicated for 5 min at 80% amplitude with a pulse cycle of 5 seconds on and 2 seconds off in a Vibra-Cell 75041 ultrasonic water bath (Bioblocks Scientic) and immediately transferred on cultured SH-SY5Y and incubated 18 h. Cells were further washed with PBS and 0.01% trypsin (3 times) in order to eliminate non-internalized fibrils, prior to be treated with 15 nM of tolytoxin or methanol for 18 h in parallel to control cells. These cells were used as donor population in co-culture experiments whereas GFP transfected SH-SY5Y cells were used as acceptor population (Supplementary Fig. S4d). After cells were co-cultured for 24 h, they were fixed with 4% PFA 20 min at RT and stained for WGA Alexa Fluor©-647 and DAPI and then mounted. Acceptor cells received α-syn fibrils and the number of α-syn puncta per acceptor cell were then quantified in parallel to secretion test.

**Supplementary Table-1.** Percentage of TNT-connected cells for both cell types. Mean percentage of TNT-connected cells and the standard error of the mean for two sets of TNT counting experiments (three independent experiments for one set of experiment) performed for SW13 and one set of experiment (three independent experiments) performed for SH-SY5Y cells.

| **SW13** | **TNT-connected cells (%)** |
|---|---|
| Control | 48.37 ± 4.168 |
| 0.5 nM methanol | 46.8 ± 3.998 |
| 0.5 nM tolytoxin | 45.17 ± 4.945 |
| 1.5 nM methanol | 53.9 ± 5.376 |
| 1.5 nM tolytoxin | 40.69 ± 4.251 |
| 3 nM methanol | 50.33 ± 3.604 |
| 3 nM tolytoxin | 29.23 ± 4.371 |
| | |
| Control | 44.24 ± 3.659 |
| 5 nM methanol | 39.8 ± 5.106 |
| 5 nM tolytoxin | 20.19 ± 6.75 |
| 10 nM methanol | 35 ± 9.68 |
| 10 nM tolytoxin | 17.55 ± 5.044 |
| 20 nM methanol | 33.4 ± 7.891 |
| 20 nM tolytoxin | 15.75 ± 5.429 |
| | |
| **SH-SY5Y** | **TNT-connected cells c (%)** |
| Control | 43.16 ± 2.148 |
| 3 nM methanol | 44 ± 5.494 |
| 3 nM tolytoxin | 40.16 ± 4.018 |
| 5 nM methanol | 39.23 ± 3.085 |
| 5 nM tolytoxin | 37.61 ± 3.656 |
| 15 nM methanol | 46.37 ± 2.822 |
| 15 nM tolytoxin | 32.53 ± 2.489 |

**Supplementary Figure S1.** Comparison of tolytoxin and luminaolide B biosynthesis gene clusters.

**a**) Gene cluster conservation and rearrangement in four strains producing tolytoxin or luminaolide B. Named accordingly to the reference gene cluster of *Scytonema* sp. PCC 10023, the tolytoxin gene clusters include one acyltransferase (*ttoA*), one thioesterase (*ttoH*) and four to five *trans* AT-PKS and (*tto C-F*) indicated in blue, one cytochrome P450 gene (*ttoG*) in green, permease and transporter (brown), transposase (yellow), and other genes with unknown function (white). The grey shadowed areas link conserved genes between the four pathways according to phylogeny of the KS genes, which highlight the rearrangement of the cluster from one strain to another. **b**) Spectrometry and purity check of tolytoxin from *Planktothrix paucivesiculata* PCC 8926, Mass spectrum of the peak at 17.64 min from PCC 8926 (*m*/*z* 872.51 $[M+Na]^+$, *m*/*z* 832.52 $[M+H-H_2O]^+$, *m*/*z* 814.51 $[M+H-2H_2O]^+$). **c)** Purity of the tolytoxin extracted from PCC 8926 used for this study. $^1$H NMR data of tolytoxin (upper) and PCC 8926-Fr.3 (lower) in acetone-$d_6$ at 298 K.

## Supplementary Figure-S1

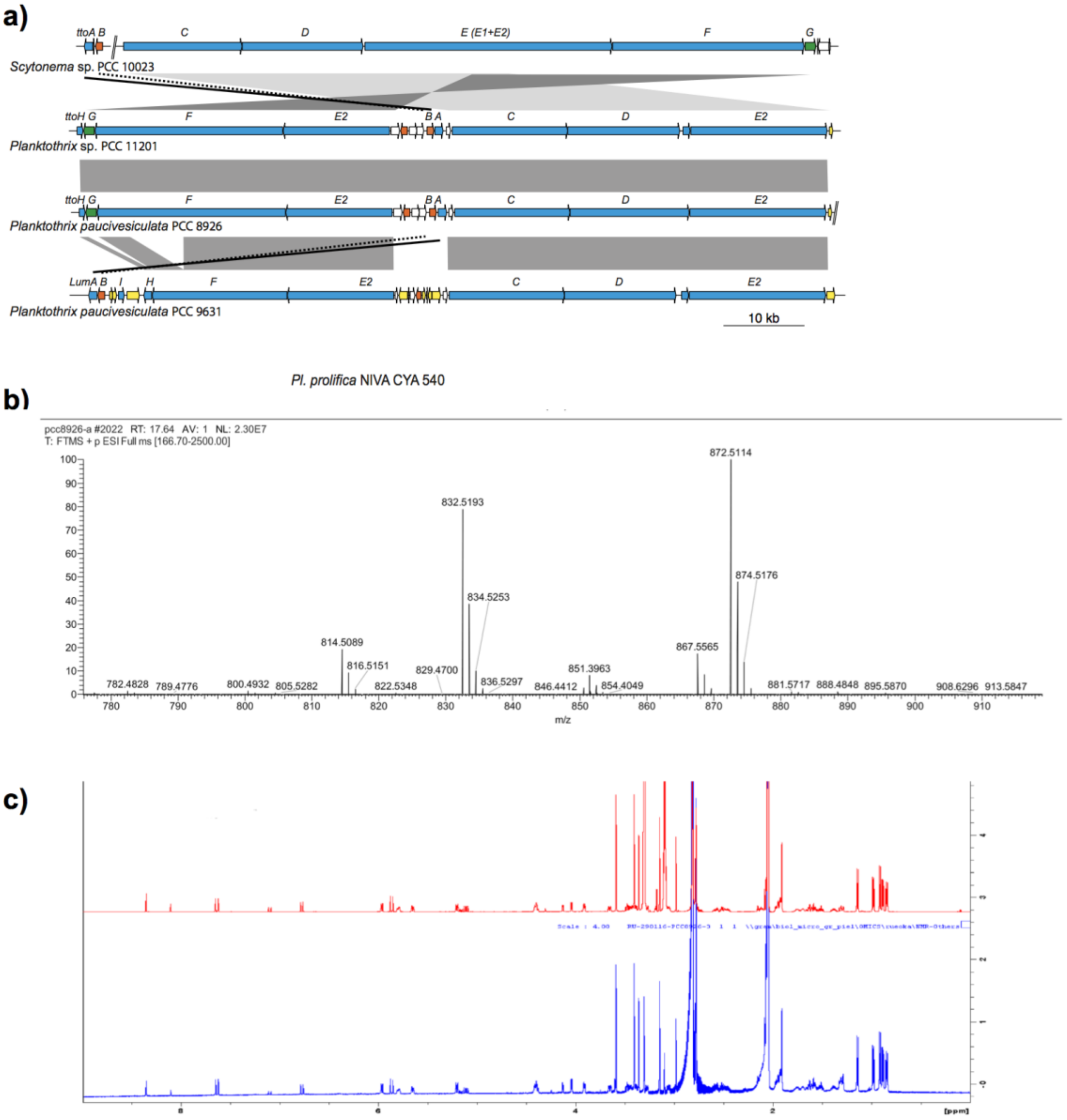

**Supplementary Figure S2.** Increase in Pan-actin / F-actin ratio in the presence of tolytoxin, TNTs without cargo and having either mitochondria or α-syn in Z-stacks. Effect of tolytoxin on actin cytoskeleton in SKOV-3 cells

**a)** F- and Pan-actin labeling for Control, 100 nM Me-control and 100 nM tolytoxin treated SW13 and SHSY-5Y cells. Arrows indicate increased G-actin labelling. Scale bar: 10 µm. **b)** upper graph: Pan Actin/F actin ratio in SW13 cells; Control: 0.28 ± 0.01, Me-Control: 0.58 ± 0.02, 3 nM tolytoxin: 1.29 ± 0.17 (p=0.06 for control *versus* 3 nM Me-control, p<0.0001 for control *versus* 3 nM tolytoxin, p<0.0001 for 3 nM Me-control *versus* 3 nM tolytoxin). Lower graph: Pan Actin/F actin ratio in SH-SY5Y cells; Control: 0.55 ± 0.01, Me-Control: 0.6 ± 0.02, 15 nM tolytoxin: 0.88 ± 0.04 (p=0.68 for control *versus* 15 nM Me-control, p<0.0001 for control *versus* 15 nM tolytoxin, p<0.0001 for 15 nM Me-control *versus* 15 nM tolytoxin). Data were collected from three independent experiments; 40 cells were analysed per condition. **c)** Sequential image sections of TNTs from upper slices to bottom slice in SW13 and SHSY-5Y cells. Arrows indicate TNTs forming from upper sections and disappearing in bottom sections. Scale bar: 10 µm. **d**) Bottom and upper sections of mitochondria (MitoDsred) and WGA Alexa 488 labeled SH-SY5Y cells. White arrow indicates mitochondria inside of a TNT visible only in upper section. Selected area (indicated with white rectangle) is presented in 3D in a different angle, WGA Alexa 488 is pseudo-colored in grey (Left). Bottom and upper sections of Atto 550 tagged α-syn fibrils and WGA Alexa 488 labeling in SH-SY5Y cells. White arrow indicates α-syn inside of a TNT visible only in upper section. Selected area (indicated with white rectangle) is presented in 3D in a different angle, WGA Alexa 488 is pseudo-colored in grey in both IF and 3D representation (Right). Scale bars: 10 µm for IF images and 5 µm for 3D presentations). **e)** Orthogonal view of mitochondria transferred into the cytoplasm of an acceptor cell in SH-SY5Y co-culture. Scale bar: 10 µm. **f)** Rhodamine-phalloidin labeling in SKOV-3 cells in Control, 3 nM and 15 nM methanol or tolytoxin treated cells. Arrows indicate intact actin filaments. Scale bar: 20 µm.

**Supplementary Figure-S2**

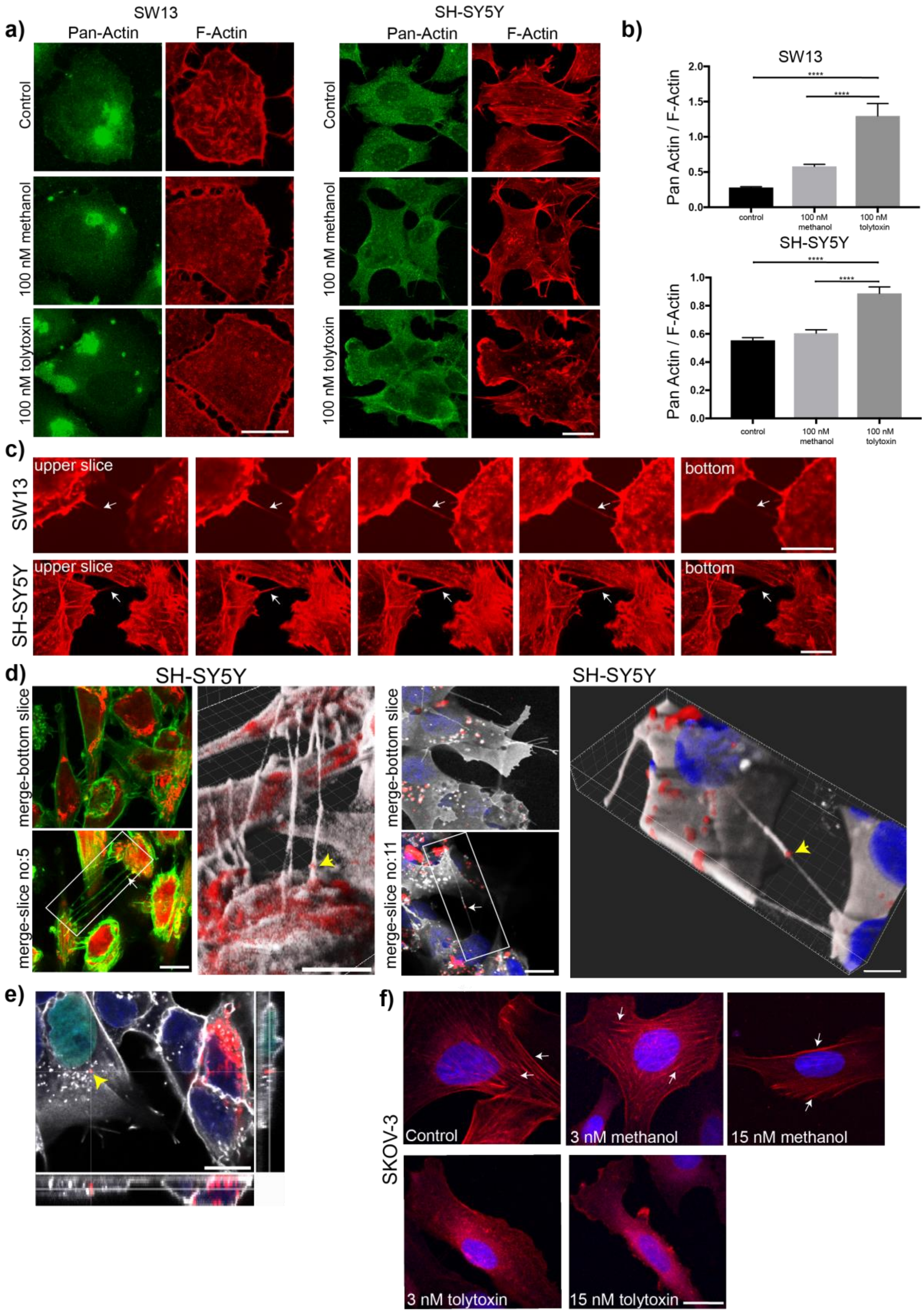

**Supplementary Figure S3.** Effect of tolytoxin on filopodia formation.

**a)** Attached filopodia detection in SW13 cells. Control, Me-Control and 3 nM tolytoxin treated cells labelled with vinculin, HCS Cell Mask$^{TM}$ Blue Stain and DAPI. **b)** Graph showing the average number of attached filopodia quantification in SW13 cells. Mean number of attached filopodia in Control: 88.32 ± 4.16, Me-control: 78.73 ± 3.17, 3 nM tolytoxin: 86.94 ± 3.08 (p=0.141 for control *versus* 3 nM Me-control, p=0.956 for control *versus* 3 nM tolytoxin, p=0.231 for 3 nM Me-control *versus* 3 nM tolytoxin). **c)** Attached filopodia detection in SH-SY5Y cells. Control, Me-Control and 15 nM tolytoxin treated SH-SY5Y cells labelled with vinculin, rhodamine phalloidine and DAPI. **d)** Graph showing the average number of attached filopodia in Control: 43.41 ± 3.89, Me-control: 57,85 ± 6.66, 15 nM tolytoxin: 39.42 ± 3.34 (p=0.09 for control *versus* 15 nM Me-control, p=0.832 for control *versus* 15 nM tolytoxin, p=0.06 for 15 nM Me-control *versus* 15 nM tolytoxin). Data were collected from three independent experiments; 40 cells were analysed per condition**.** Scale bar: 10 µm.

**Supplementary Figure-S3**

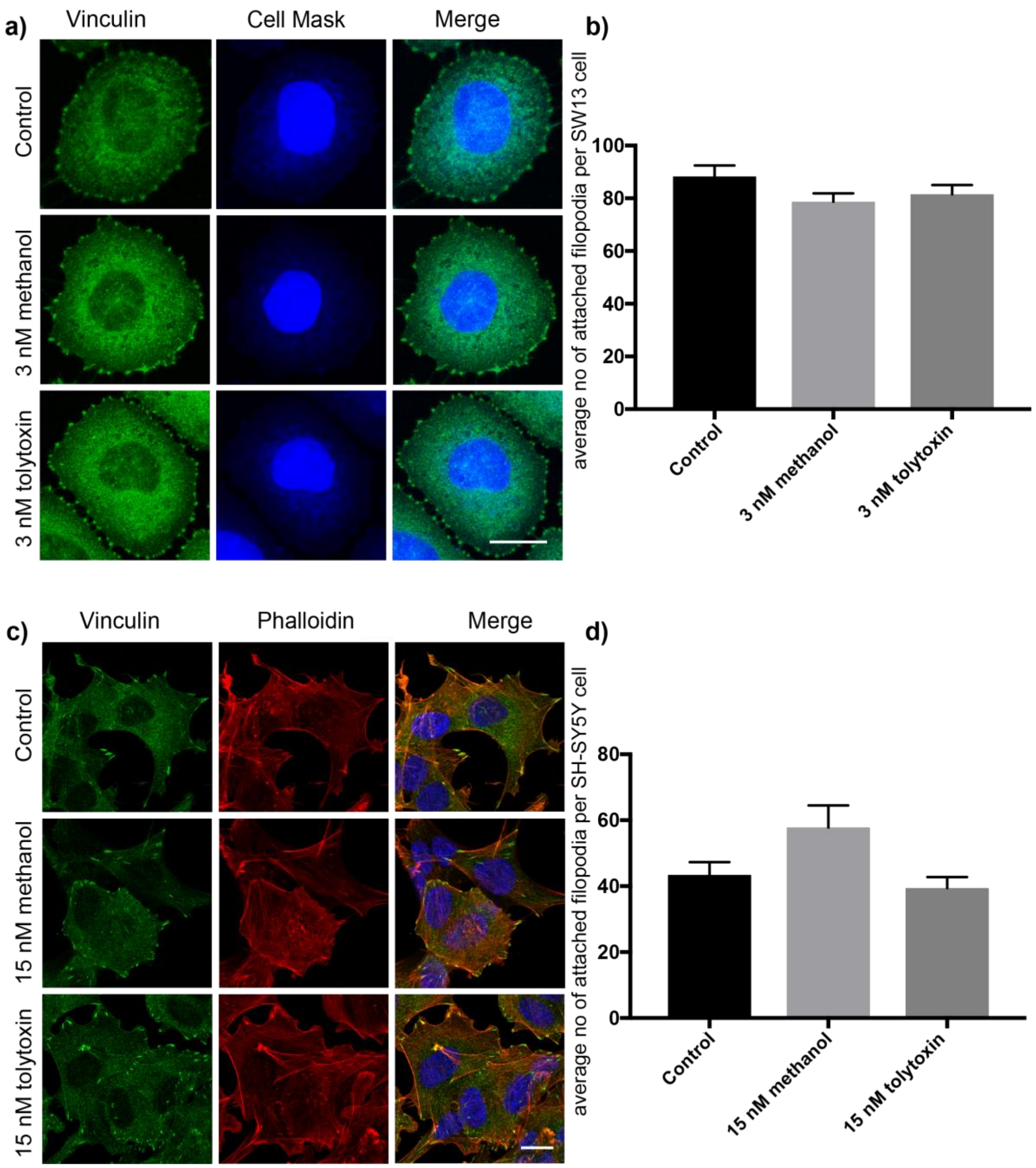

**Supplementary Figure S4.** Schematic of the experimental design of co-culture experiments. **a)** Experimental design for DiD vesicle transfer: Donor SW13 cells were either treated 18 h with 3 nM of methanol or tolytoxin or left untreated. They were labeled with vybrant DiD dye for 20 min and co-cultured with H2B-GFP transfected acceptor cells for 24 h. **b)** Experimental design for mitochondria transfer in SW13 cells: Donor cells were transfected with Mito-GFP plasmid for 24 h and treated either with 3 nM of methanol or tolytoxin or left untreated. They were then co-cultured with H2B-mCherry transfected acceptor cells for 24 h. **c)** Experimental design for mitochondria transfer in SH-SY5Y cells: Mito-Dsred expressing SH-SY5Y donor cells were treated with 15 nM of methanol or tolytoxin or left untreated. They were then co-cultured with H2B-GFP expressing acceptor cells for 24 h. **d)** Experimental design for $\alpha$-syn fibril transfer: SH-SY5Y donor cells were loaded with Atto-550 tagged $\alpha$-syn fibrils for 18h and then treated with 15 nM of methanol or tolytoxin or left untreated. They were then co-cultured with GFP transfected acceptor cells for 24 h. **e)** Secretion test: For all the co-culture experiments performed, secretion test was conducted in parallel by collecting the conditioned medium of donor cells described in A to D and transferring on top of the suitable acceptor cell for 24 h.

## Supplementary Figure-S4

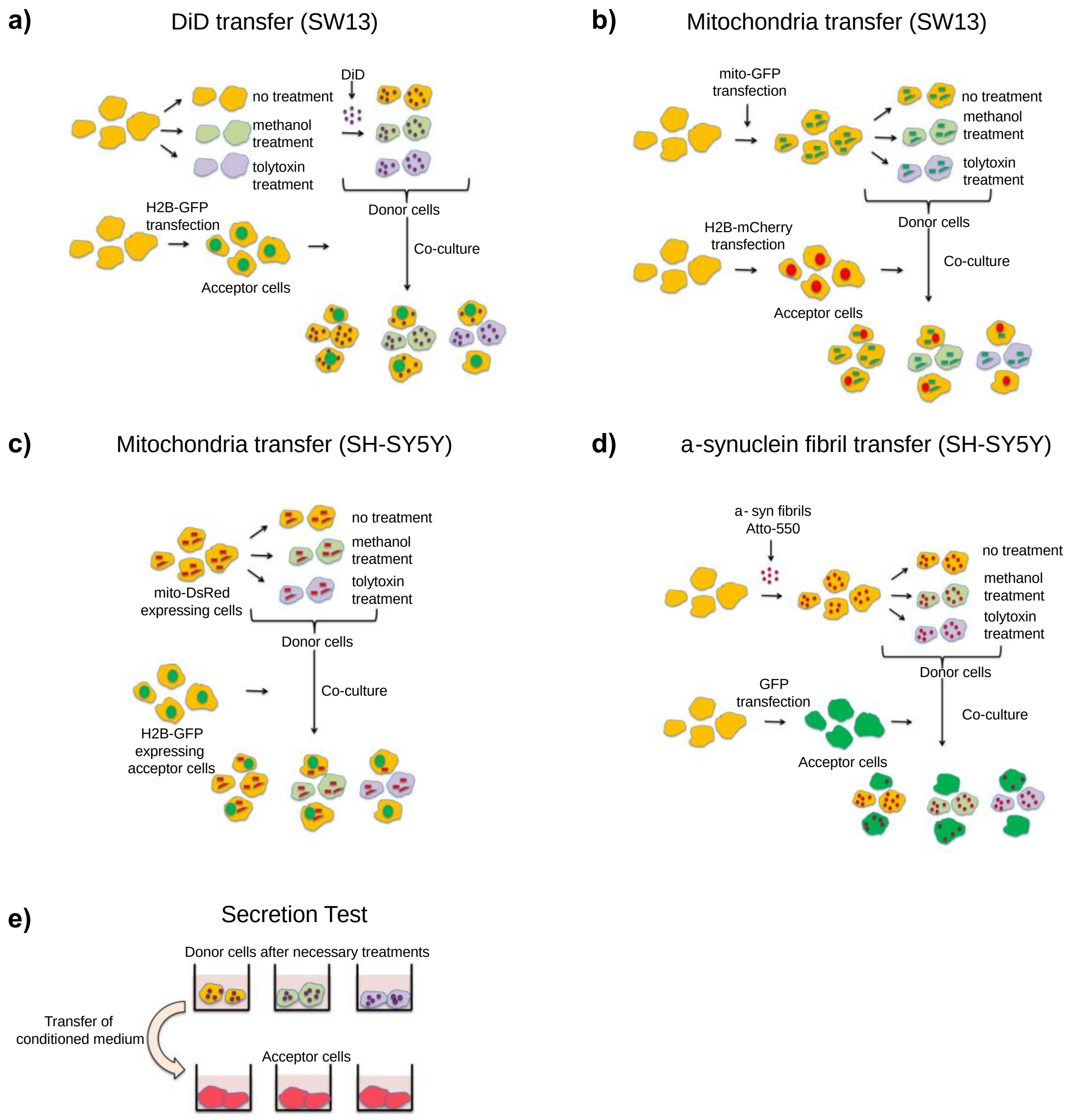

**Supplementary Figure S5.** Tolytoxin decreases the DiD labeled vesicle transfer in SW13 cells. **a)** Donor cells loaded with vybrant DiD cell labeling solution in Control, 3 nM Me-control and 3 nM tolytoxin co-cultures (left panel), acceptor cells transfected with H2B-mCherry plasmid (middle panel), donor and acceptor cells that were mixed with 1:1 ratio in different co-culture conditions (right panel). Arrows indicate DiD-positive vesicles that had been received by acceptor cells. Scale bar:10 μm. **b)** Left graph showing the percentage of DiD transfer in each co-culture condition. Mean percentage of DiD transfer in control: 63.47% ± 4.80, Me-control: 58.81% ± 4.47, 3 nM tolytoxin: 42.4% ± 5.18 (p=0.777 for Control *versus* 3 nM Me-control, p=0.0088 for Control *versus* 3 nM tolytoxin, p=0.0453 for 3 nM Me-control *versus* 3 nM tolytoxin). Right graph showing the average number of DiD vesicles per acceptor cell in each co-culture condition: Control: 12.49% ± 1,58, Me-control: 9.31% ± 0.88, 3 nM tolytoxin: 10.74% ± 1,52 (p=0.839 for Control *versus* 3 nM Me-control, p=0.585 for Control *versus* 3 nM tolytoxin, p=0.45 for 3 nM Me-control *versus* 3 nM tolytoxin). Data were collected from three independent experiments; 50 cells were analysed per condition**.**

**Supplementary Figure-S5**

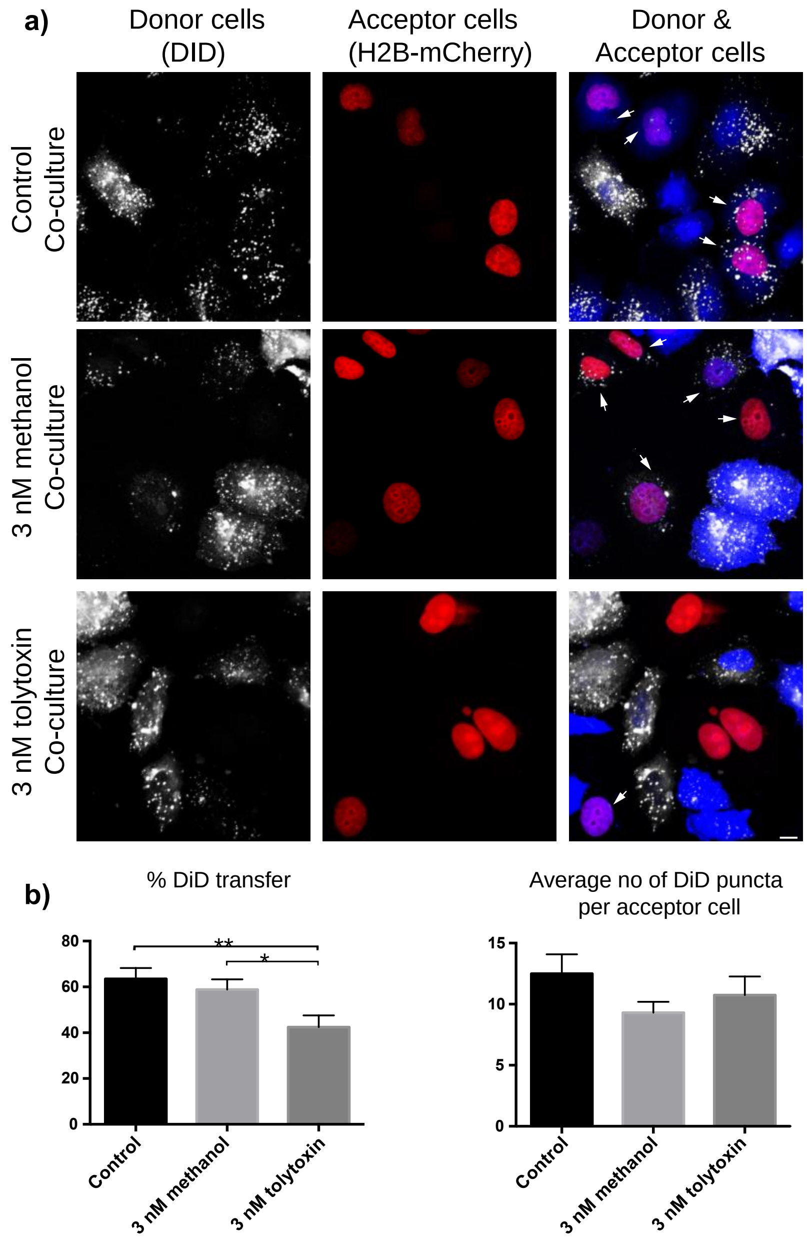


240
